# Exploring the pathogenesis, biomarkers, and potential drugs for type 2 diabetes mellitus and acute pancreatitis through a comprehensive bioinformatic analysis

**DOI:** 10.3389/fendo.2024.1405726

**Published:** 2024-11-20

**Authors:** Lei Zhong, Xi Yang, Yuxuan Shang, Yao Yang, Junchen Li, Shuo Liu, Yunshu Zhang, Jifeng Liu, Xingchi Jiang

**Affiliations:** ^1^ Department of General Surgery, The First Affiliated Hospital of Dalian Medical University, Dalian, Liaoning, China; ^2^ Department of Plastic Surgery, The First Affiliated Hospital of Dalian Medical University, Dalian, Liaoning, China; ^3^ Department of Endocrinology and Metabolic Diseases, The First Affiliated Hospital of Dalian Medical University, Dalian, Liaoning, China

**Keywords:** acute pancreatitis, type 2 diabetes mellitus, molecular docking, machine learning, biomarker

## Abstract

**Background:**

Type 2 diabetes mellitus (T2DM) is a chronic metabolic disease that accounts for > 90% of all diabetes cases. Acute pancreatitis (AP) can be triggered by various factors and is a potentially life-threatening condition. Although T2DM has been shown to have a close relationship with AP, the common mechanisms underlying the two conditions remain unclear.

**Methods:**

We identified common differentially expressed genes (DEGs) in T2DM and AP and used functional enrichment analysis and Mendelian randomization to understand the underlying mechanisms. Subsequently, we used several machine learning algorithms to identify candidate biomarkers and construct a diagnostic nomogram for T2DM and AP. The diagnostic performance of the model was evaluated using ROC, calibration, and DCA curves. Furthermore, we investigated the potential roles of core genes in T2DM and AP using GSEA, xCell, and single-cell atlas and by constructing a ceRNA network. Finally, we identified potential small-molecule compounds with therapeutic effects on T2DM and AP using the CMap database and molecular docking.

**Results:**

A total of 26 DEGs, with 14 upregulated and 12 downregulated genes, were common between T2DM and AP. According to functional and DisGeNET enrichment analysis, these DEGs were mainly enriched in immune effector processes, blood vessel development, dyslipidemia, and hyperlipidemia. Mendelian randomization analyses further suggested that lipids may be a potential link between AP and T2DM. Machine learning algorithms revealed ARHGEF9 and SLPI as common genes associated with the two diseases. ROC, calibration, and DCA curves showed that the two-gene model had good diagnostic efficacy. Additionally, the two genes were found to be closely associated with immune cell infiltration. Finally, imatinib was identified as a potential compound for the treatment of T2DM and AP.

**Conclusion:**

This study suggests that abnormal lipid metabolism is a potential crosstalk mechanism between T2DM and AP. In addition, we established a two-gene model for the clinical diagnosis of T2DM and AP and identified imatinib as a potential therapeutic agent for both diseases.

## 1 Introduction

The incidence of acute pancreatitis (AP), an inflammatory disease, varies based on the geographic location, with the annual incidence being approximately 34 cases per 100,000 individuals in the general population worldwide (1). Patients with moderately severe or severe AP have pancreatic necrosis or pancreas failure and an extended hospital stay (2). Exocrine pancreatic insufficiency, walled-off pancreatic necrosis, and recurring AP are long-term sequelae in approximately 20% of the patients (3, 4). Although the overall morbidity and mortality rates of AP have decreased as a result of advances in aggressive fluid resuscitation, supportive treatment, and early risk stratification, the mechanisms and risk factors that underlie these improvements and affect intermediate and long-term outcomes remain unknown (5). Therefore, a deeper understanding of the pathological mechanisms underlying AP is necessary to identify novel biomarkers for early diagnosis and treatment.

Diabetes mellitus (DM) is a common metabolic condition worldwide. According to the World Health Organization (WHO), an estimated 422 million people have DM worldwide, with type 2 diabetes mellitus (T2DM) being the most prevalent (6). Studies have shown that pancreatic fat deposition can lead to long-term exposure of pancreatic beta cells to high levels of fatty acids and triglycerides, resulting in abnormal insulin secretion signaling and an increased risk of T2DM (7). A meta-analysis of seven observational studies showed that individuals with T2DM had an 84% higher risk of developing AP than those without DM (8). In addition, two recent meta-analyses have shown that approximately 23% of patients with AP may develop DM within 3 years of discharge, highlighting the high incidence rate and chronic nature of AP-related DM (9, 10). These findings indicate the presence of a common pathological mechanism between AP and T2DM.

Although earlier studies have provided valuable insights into the relationship between T2DM and AP, more comprehensive studies are required to address existing knowledge gaps. In recent years, integrated bioinformatic analysis has been used to identify disease-associated novel genes that may be used as diagnostic and prognostic biomarkers. Unknown exists regarding the shared diagnosis and related genes between T2DM and AP. Therefore, this study aimed to identify biomarkers for AP and T2DM through bioinformatic analysis. The findings may provide a theoretical foundation for developing novel diagnostic and therapeutic strategies for the two conditions.

## 2 Methods

### 2.1 Data collection from GEO databases

The datasets analyzed in this study were obtained from the GEO database, which included T2DM datasets GSE20966 (11), GSE25724 (12), and one AP dataset, GSE194331 (13). The T2DM datasets GSE20966 and GSE25724 were merged for analysis by the “sva” package (14). The |log2 Fold change (FC)| > 0.585 and adjust *p <*0.05 were set as the criteria for identifying differentially expressed genes (DEGs) of AP and T2DM using the “limma” package (15). Finally, the Venn map was applied to select their common genes.

### 2.2 Analysis of functional enrichment

The STRING database (https://cn.string-db.org/) was used to investigate protein interactions, with the validity of such interactions being determined by a composite score greater than 0.15 (16). Meanwhile, GeneMANIA database (https://genemania.org/) prioritized genes for functional tests (17). Functional enrichment studies were performed by Metascape database (https://metascape.org/), which was designed to provide an extensive resource for annotating and analyzing gene lists to investigate the biological roles and routes implicated in certain genes (18).

### 2.3 Mendelian randomization analysis

Mendelian randomization analysis was conducted using the R package “TwoSampleMR (v.0.5.6)” and strictly adhered to the three fundamental assumptions of Mendelian randomization (MR): (I) There exists a strong association between the instrumental variables (IVs) and the exposure; (II) The IVs are not associated with potential confounding factors; (III) The IVs influence the outcome solely through the exposure. The inverse-variance weighted (IVW) method was employed as the primary analytical approach (19), complemented by four additional methods. The datasets utilized in this study were sourced from the IEU OPEN GWAS (https://gwas.mrcieu.ac.uk/). Specifically, the GWAS for HDL-C included 94,595 participants and a total of 2,418,527 single nucleotide polymorphisms (SNPs); the GWAS for LDL-C comprised 173,082 participants and 2,437,752 SNPs; the GWAS for triglycerides (TG) involved 177,861 participants and 2,439,433 SNPs; and the GWAS for apolipoprotein A-I (APOA-I) encompassed 393,193 participants and 12,321,875 SNPs. The threshold for instrumental variable selection was set at *p*<5E-08, r^2^<0.001, with a clumping distance of 10,000 kb. Heterogeneity testing was performed using the “mr_heterogeneity” function, and horizontal pleiotropy testing was conducted via the “mr_pleiotropy_test” function and “MR-PRESSO” R package (19), and sensitivity analysis was executed using the “leave-one-out” method.

### 2.4 Using machine learning to screen characteristic genes

LASSO, RF, and SVM-RFE were performed to filter genes in both AP and T2DM, respectively. To mitigate overfitting among genes, LASSO regression analysis was applied, followed by cross-validation by the package “glmnet” (20, 21). The “Random Forest” R software was used to conduct RF (22). Genes with importance > 2 in AP samples and importance > 1 in T2DM samples were selected as feature genes. In addition, SVM-RFE was performed using the R package “e1071”, aiming to optimize the learning performance by minimizing the empirical error (23). The hub genes for the following studies were then selected from the intersection of the three subsets. The intersection of the core genes of AP and T2DM was used as the biomarkers for the two diseases. The expression levels of the core genes in the disease and control groups are shown in boxplots (wilcox test).

### 2.5 Construction of the nomogram

The ROC curves were computed using the “pROC” program in order to assess the predictability of the model (24). Using the “rms” package, a nomogram incorporating model genes was created (25). In addition, the model’s predicted accuracy was assessed using the DCA and calibration curves (26).

### 2.6 Validation of core genes

We downloaded the human T2DM dataset GSE95849 (27) to further validate the expression levels of core genes in T2DM. The wilcox test was used to compare the difference in expression of core genes between disease and control groups, with *p* < 0.05 considered statistically significant. However, due to the lack of another human AP dataset in the public database, we chose mouse dataset GSE77983 (28) for validation. We used the GEO2R (based on the R package “limma”) online tool of the GEO database to analyze GSE77983 to verify the differential expression of core genes between the AP and control groups.

### 2.7 Cell culture and quantitative real-time PCR analysis

The mouse pancreatic acinar cell line 266-6 (ATCC; VA, USA), were cultured in DMEM with 10% fetal bovine serum, 100 IU/ml penicillin and 100 μg/ml streptomycin. To induce pancreatitis *in vitro*, the 266-6 cells were stimulated with 250μM Sodium taurocholate (STC) (29). Twenty-four hours later, cells were collected for the following qPCR analysis.

Total RNA from cells was extracted using Trizol reagent (Accurate Biology, Hunan), and the circRNAs were reversely transcribed using the Evo M-MLV for qPCR (Accurate Biology, Hunan). Then, cDNAs were synthesized and quantified via SYBR Green Pro Taq HS (Accurate Biology, Hunan) under the following cycle scheme: 95°C for 30s, then 95°C for 5 s and 60°C for 30 s for 40 cycles. The RNA expression levels were analyzed and quantified using the ΔΔCt method, and the expression levels of the target genes were compared between the two groups using the t-test. The following primers were employed: IL-6 primer (forward: 5’-GAGAGGAGACTTCACAGAGGATACC-3’; reverse: 5’-TCATTTCCACGATTTCCCAGAGAAC-3’), IL-1β primer (forward: 5’-AGGTCGGTGTGAACGGATTTG-3’; reverse: 5’-TGAGAAGAGGCTGAGACATAGGC-3’), SLPI primer (forward: 5’-GAAGCCACAATGCCGTACTGAC-3’; reverse: 5’-GGAACAGGATTCACGCACTTGG-3’), ARHGEF9 primer (forward: 5’-GAAGCAGTGCCGAAAGAGAAGG-3’; reverse: 5’-ACGAAGCCCATCTGAAATCTGTATATG-3’), and Actin primer (forward: 5’-ACTGCCGCATCCTCTTCCTC-3’; reverse: 5’-AACCGCTCGTTGCCAATAGTG-3’).

### 2.8 Potential functions of model genes in AP and T2DM

Gene set enrichment analysis (GSEA) is utilized to elucidate the molecular mechanisms between high- and low-core gene expression samples, and results with a *p* value < 0.05 were considered significant (30). Meanwhile, the xCell method was also used to assess the correlation between significantly different enriched immune cell types and characteristic genes, which was considered to be correlated at *p* < 0.05.

### 2.9 Construction of ceRNA network

The TargetScan, miRDB, and miRanda databases were used to anticipate miRNA-mRNA pairs in order to identify the ceRNA network that might be influenced by model genes. Genes that were simultaneously listed in three databases were the only ones that were thought to be possible mRNA targets for further research. To predict miRNA-lncRNA pairs, the spongeScan database was used. At last, the ceRNA network could be seen using Cytoscape (31). Meanwhile, the Human Protein Atlas (HPA: https://www.proteinatlas.org/) was utilized to examine the model genes’ immunofluorescence and single-cell type atlases.

### 2.10 Identifying potential small molecule compounds for the treatment of AP and T2DM

The CMap database (https://clue.io/) can link diseases, genes and drugs based on similar or opposite gene expression profiles (32). Commonly upregulated DEGs in AP and T2DM were entered into the CMap database to identify potential small molecule compounds for the treatment of AP and T2DM. Then, the protein structures of the feature genes were obtained from the PDB database, and the AutoDock tool was applied to calculate the protein hydrogenation and charge. PubChemdatabase to download the chemical structure of the drug’s active ingredient. The AutoDock tool is used to check the charge balance and rotatable bonds of tiny molecules. To generate docking energy, AutoDock Vina runs docking simulations. Finally, PyMol software was used to check the docking complex.

## 3 Results

### 3.1 Identification and analysis of DEGs in AP and T2DM

**Figure 1:**
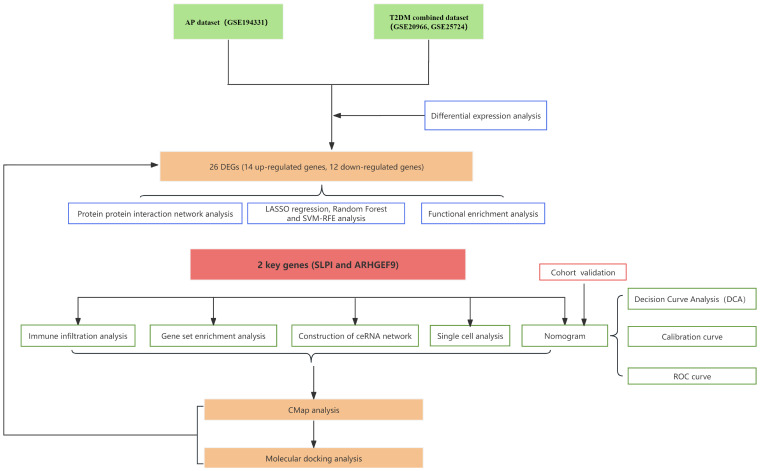
The process of data analyzing in this study.

**Figure 2:**
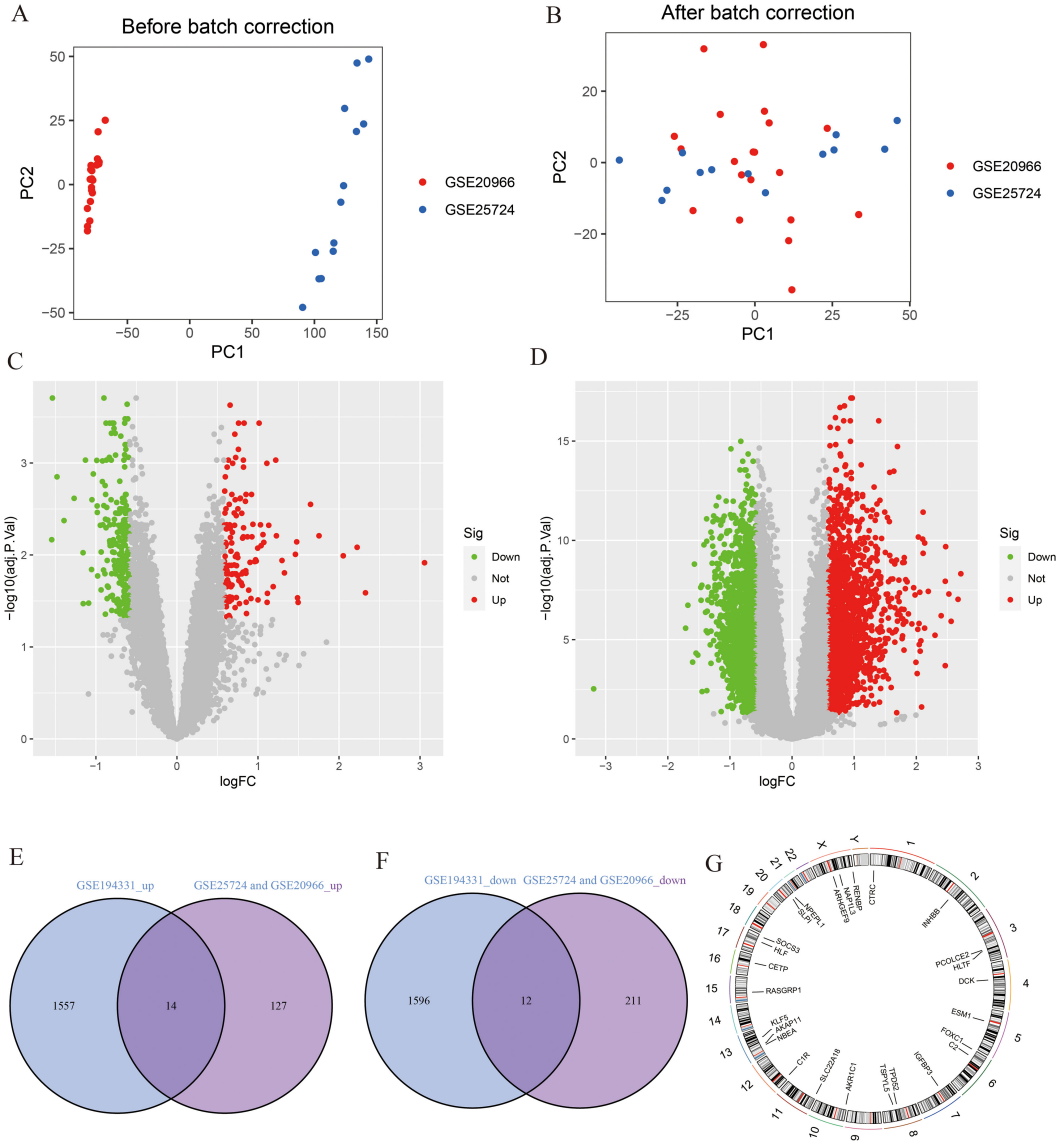
Identification of DEGs. **(A, B)** PCA analysis before and after merging of T2DM datasets; **(C)** The volcano plot for T2DM (|log2 FC| > 0.585 and adjust *p <*0.05); **(D)** The volcano plot for AP (|log2 FC| > 0.585 and adjust *p <*0.05); **(E)** The intersection of AP up-regulated DEGs and T2DM up-regulated DEGs; **(F)** The intersection of AP down-regulated DEGs and T2DM down-regulated DEGs; **(G)** The location of DEGs on chromosomes.

A flowchart demonstrating the study protocol is presented in **Figure 1**. Initially, we merged two T2DM datasets and corrected batch effects using the “sva” software package. As shown in **Figures 2A, B**, the differences between batches were effectively eliminated after data normalization, indicating that the two datasets could be merged. The volcano map presented in **Figures 2C, D** shows DEGs in T2DM and AP (|log2 FC| > 0.585 and adjusted *p* < 0.05). Venn diagram, the up-regulated and down-regulated genes of the two datasets were crossed, respectively, and 14 up-regulated DEGs and 12 down-regulated DEGs were obtained (**Figures 2E, F**). **Figure 2G** shows the locations of these common DEGs on chromosomes.

### 3.2 PPI network and functional enrichment analyses of DEGs

**Figure 3:**
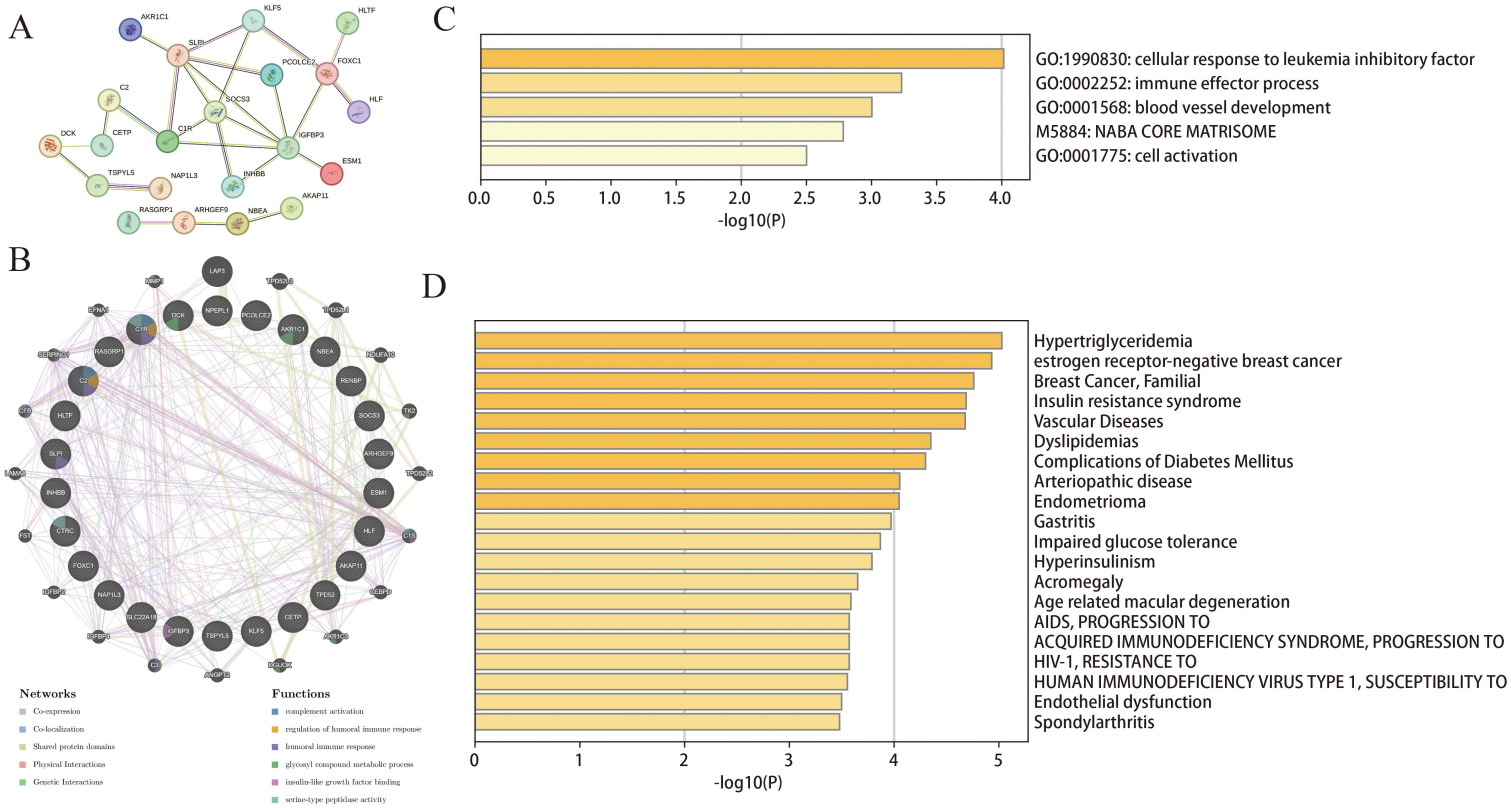
Functional enrichment analysis of DEGs. **(A)** PPI of the DEGs; **(B)** The GeneMANIA analysis for DEGs; **(C, D)** Functional and DisGeNET enrichment analyses by the Metascape database.

The 26 common DEGs were imported into the STRING database to construct a PPI network (**Figure 3A**). Subsequently, GeneMANIA was then used to further analyze DEGs for co-localization, co-expression, and shared protein domains (**Figure 3B**). The genes in the PPI network were mainly enriched in glycosyl compound metabolism, insulin-like growth factor binding, and complement activation. Furthermore, we used Metascape to determine biological processes and pathways related to the DEGs. According to the results, the DEGs were involved in immune effector processes and blood vessel development (**Figure 3C**) and were closely related to conditions such as hypertriglyceridemia and dyslipidemia (**Figure 3D**).

### 3.3 Mendelian randomization analysis

MR analysis was performed to assess the potential relationship between dyslipidemia and T2DM or AP (**Supplementary Figures S1A, B**). IVW analysis showed that HDL-C and Apoa-I exhibited significant protective effects against both T2DM (*P* = 1.57E-09 and 0.0007, respectively) and AP (*P* = 0.0269 and 0.0232, respectively). Notably, TG (*P* = 0.0001) were identified as a significant risk factor for T2DM. These results remained consistent in most models, demonstrating their robustness. However, some degree of heterogeneity was observed (**Supplementary Table S1**). To address this issue, we used a random-effect model to minimize potential biases and errors. In addition, we used the MR-PRESSO method to identify and eliminate potential outliers, ensuring non-pleiotropy and the accuracy of the results. Finally, we validated the sensitivity of the results using the leave-one-out test (**Supplementary Figures S1C–G**). Altogether, the results suggested that dyslipidemia may be a common underlying mechanism of T2DM and AP.

### 3.4 Selection of characteristic genes using machine learning algorithms

**Figure 4:**
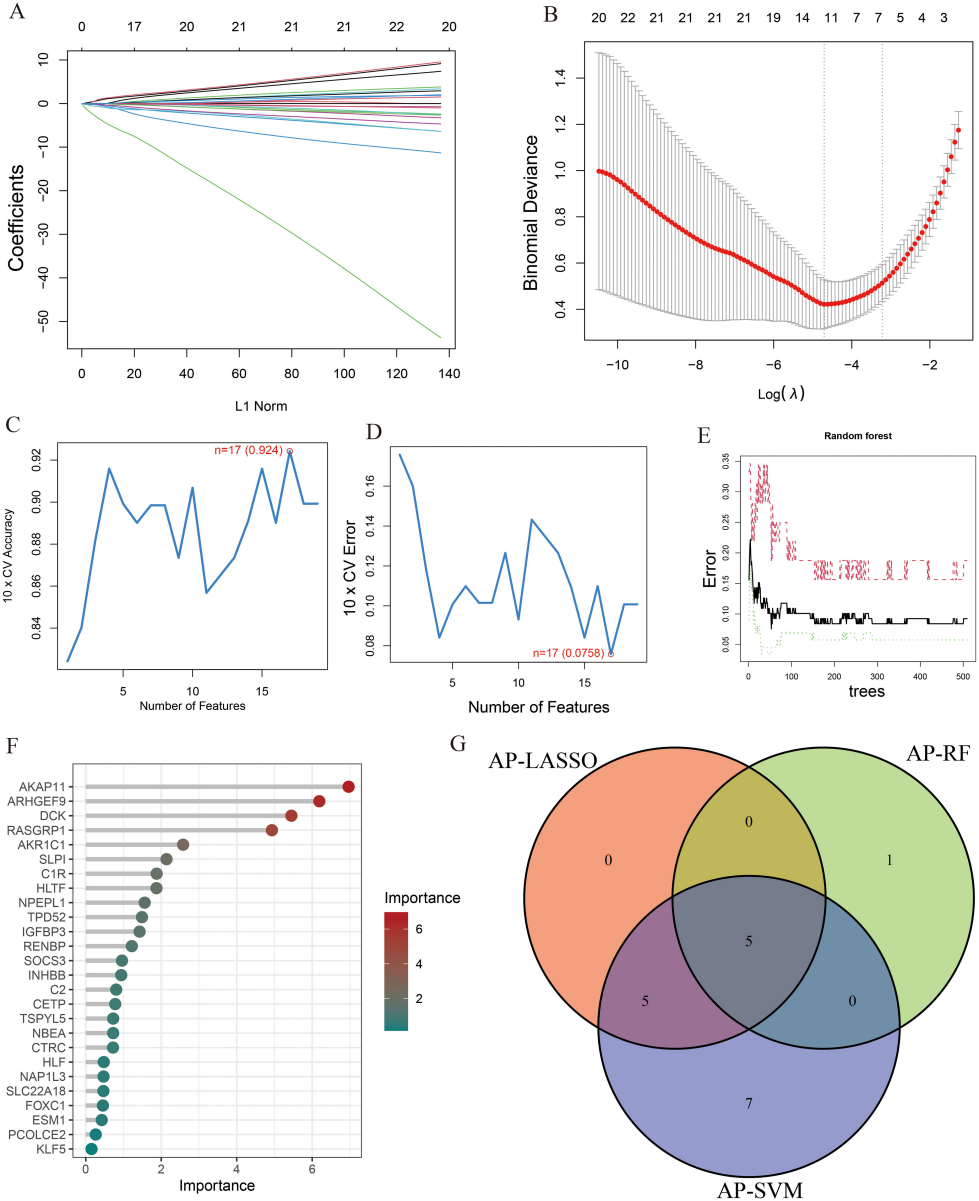
The characteristic genes of AP were screened by machine learning method. **(A)** LASSO regression analysis and **(B)** cross-validation for identifying key genes and assessing partial likelihood deviance; **(C, D)** Seventeen characteristic genes found by SVM-RFE; **(E, F)** RF ranked the importance of all genes to get 6 genes with scores for importance greater than 2; **(G)** The Venn diagram exhibiting the intersection of three machine learning models.

To identify key genes associated with the development of both AP and T2DM, we constructed three machine-learning models based on the 26 DEGs. In AP samples, ten key genes were identified using the LASSO regression (**Figures 4A, B**). Seventeen genes extracted from these genes by the SVM-RFE were identified as the best genes for AP patients (**Figures 4C, D**). According to the RF, six genes with importance greater than 2 were included in the subsequent analysis (**Figures 4E, F**). Then, five AP characteristic genes were screened out by the Venn diagram (**Figure 4G**).

**Figure 5:**
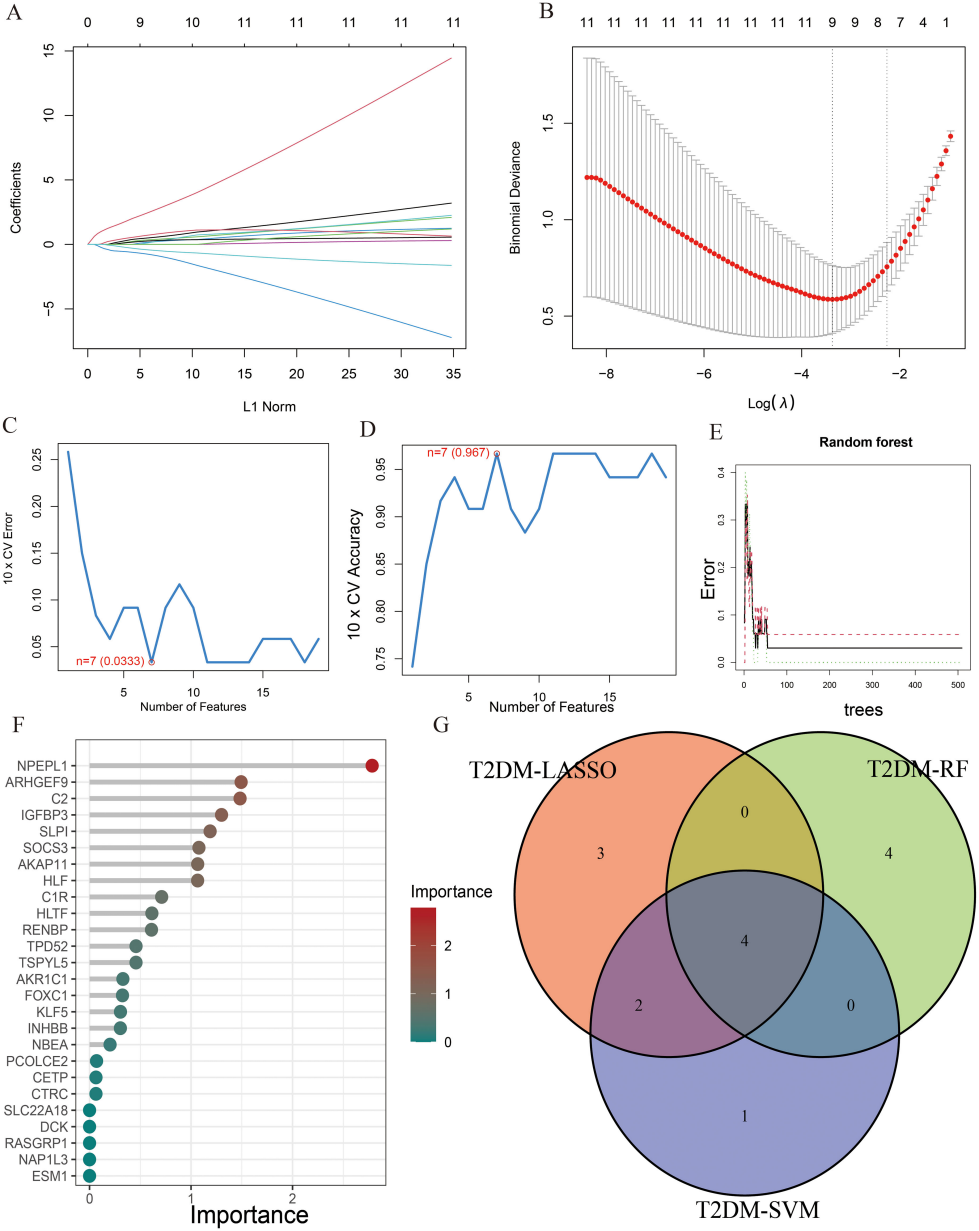
The characteristic genes of T2DM were screened by machine learning method. **(A)** LASSO regression analysis and **(B)** cross-validation for identifying key genes and assessing partial likelihood deviance; **(C, D)** Seven characteristic genes found by SVM-RFE; **(E, F)** RF ranked the importance of all genes to get 8 genes with scores for importance greater than 1; **(G)** The Venn diagram exhibiting the intersection of three machine learning models.

Similarly, the LASSO regression has shown eleven genes as potential indicators for diagnosis (**Figures 5A, B**). Using the SVM-RFE, seven genes were identified from these genes as potential biomarkers (**Figures 5C, D**). Eight genes with importance higher than 1 were included in the subsequent analysis based on the RF (**Figures 5E, F**). A Venn diagram was constructed to intersect these three gene sets, resulting in the identification of 4 key genes associated with T2DM (**Figure 5G**). Among the key genes identified in AP and T2DM, we found two common genes, SLPI and ARHGEF9, at the intersection of the Venn diagrams (**Supplementary Figure S2**). These two genes may serve as a link between AP and T2DM, playing a key role in the development of both conditions.

### 3.5 Development of a diagnostic model and assessment of its predictive efficacy

**Figure 6:**
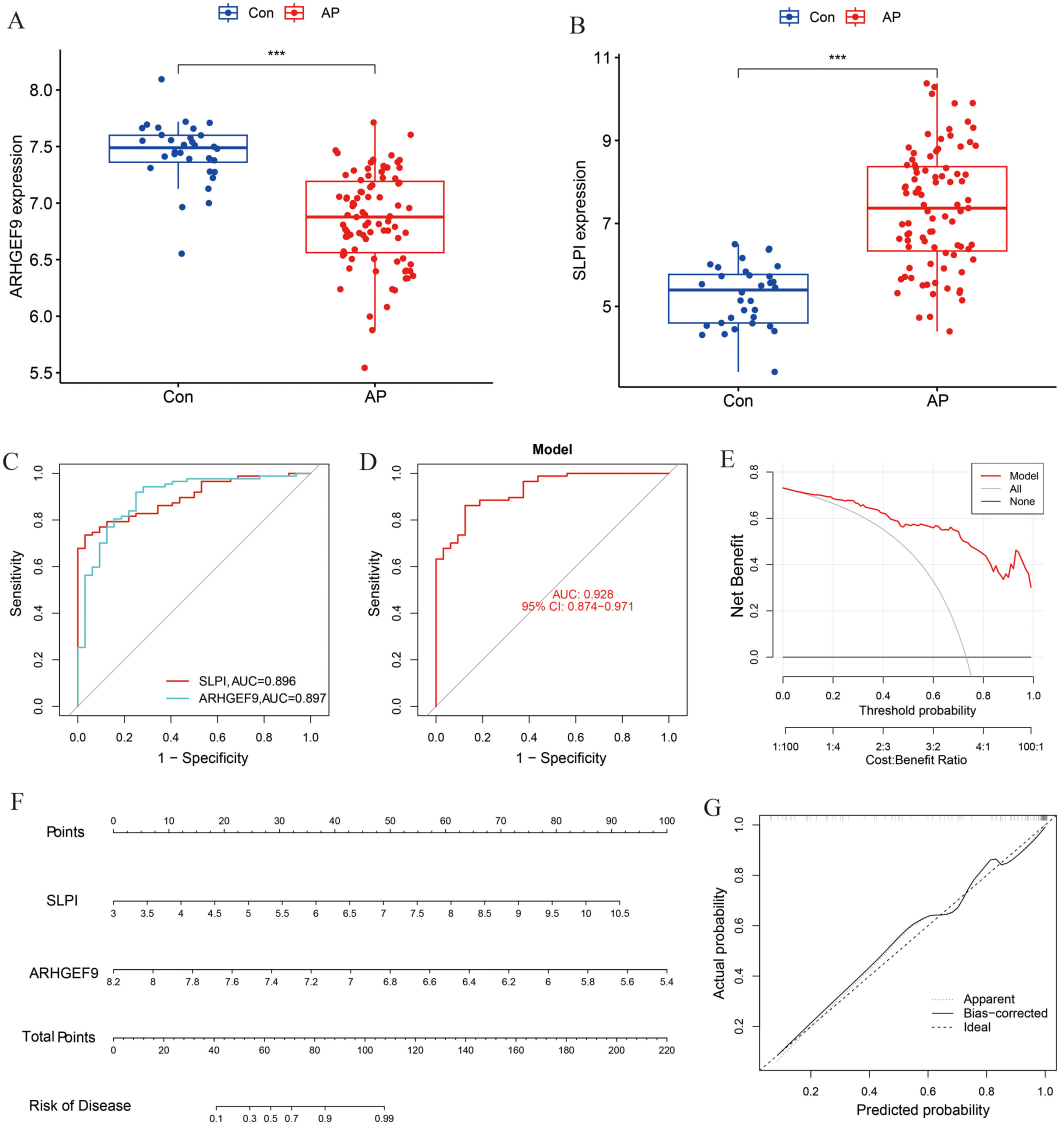
Diagnostic effect of the two-gene model on AP. Box plots showed the expression difference in **(A)** ARHGEF9 and **(B)** SLPI between AP and normal samples, ***P < 0.001; **(C)** ROC curve of diagnostic performance of ARHGEF9 and SLPI for AP; **(D)** ROC curve of the two-gene model for AP; **(E)** DCA curve of the model; **(F)** Nomogram for forecasting AP risk; **(G)** The calibration curve of nomogram model prediction in AP.

A box plot was constructed to demonstrate the expression levels of the two key genes in the disease and control groups (**Figures 6A, B**). Specifically, the expression of ARHGEF9 was significantly lower in the AP group than in the control group, whereas the expression of SLPI showed the opposite trend (*p* < 0.001). As shown in the ROC curve in **Figure 6C**, both SLPI and ARHGEF9 had high diagnostic value in AP. The AUC value of the 2-gene prediction model was 0.928, which demonstrated the high diagnostic value of both genes (**Figure 6D**). In addition, the DCA curve of the two genes showed a better overall clinical benefit than if none or all of the tests were used for diagnosing AP (**Figure 6E**). Furthermore, we developed a nomogram to assess the possible risks associated with AP (**Figure 6F**). **Figure 6G** shows that there was a minimal difference between the actual and anticipated risk for AP as indicated by the calibration curve.

**Figure 7:**
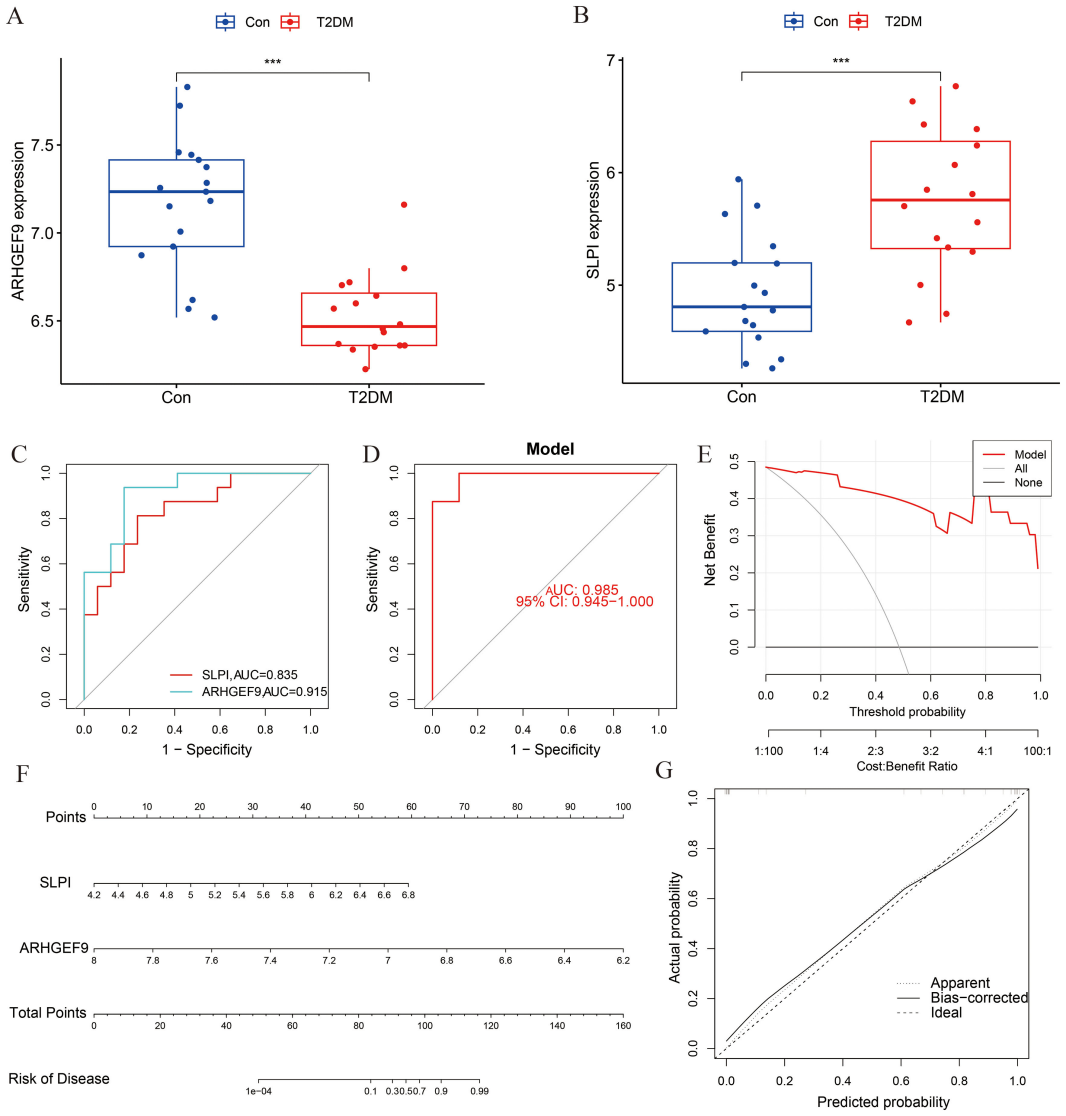
Diagnostic effect of the two-gene model on T2DM. Box plots showed the expression difference in **(A)** ARHGEF9 and **(B)** SLPI between T2DM and normal samples, ***P < 0.001; **(C)** ROC curve of diagnostic performance of ARHGEF9 and SLPI for T2DM; **(D)** ROC curve of the two-gene model for T2DM; **(E)** DCA curve of the model; **(F)** Nomogram for forecasting T2DM risk; **(G)** The calibration curve of nomogram model prediction in T2DM.

The expression levels of ARHGEF9 and SLPI in the T2DM group were consistent with those in the AP group (**Figures 7A, B**). The ROC curve showed that both SLPI and ARHGEF9 had high diagnostic value in T2DM (**Figure 7C**). The AUC value of the 2-gene model was 0.985, which emphasized the diagnostic value of the two genes (**Figure 7D**). Furthermore, the DCA curve of the two genes (**Figure 7E**) showed a better overall clinical benefit than if all or none of the tests were used for diagnosing T2DM. Subsequently, a nomogram to gauge the possible harm that T2DM individuals could cause (**Figure 7F**). The calibration curve indicates that our nomogram also has a good predictive value for T2DM (**Figure 7G**).

### 3.6 Validation of the two key genes associated with T2DM and AP

The GSE95849 dataset was used to validate the expression levels of SLPI and ARHGEF9 in T2DM. The expression of SLPI was higher in the T2DM group than in the control group, whereas that of ARHGEF9 was significantly lower in the T2DM group than in the control group (**Supplementary Figures S3A, B**). These results were consistent with those observed in the training set. Owing to the lack of another suitable human AP dataset, we analyzed the mouse AP dataset GSE77983 using the GEO2R tool to validate the expression levels of the two key genes in AP. As shown in **Supplementary Figures S3C, D**, the expression of ARHGEF9 was lower in the AP group than in the control group, whereas that of SLPI showed the opposite trend (*p* < 0.05). These results were consistent with those observed in the training set.

To additionally verify the expression levels of the two genes in AP, we developed a cell model of pancreatitis by stimulating the mouse pancreatic acinar cell line 266-6 with STC. As shown in **Supplementary Figure S3E**, the expression levels of IL-6 and IL-1β were significantly higher in the model group than in the control group, indicating that the AP model was successfully established. Subsequently, we evaluated the expression levels of SLPI and ARHGEF9 in the cells. The results showed that SLPI was upregulated in the AP group (*p* < 0.05), which is consistent with the results observed in the training set. However, no significant difference in ARHGEF9 expression was observed between the two groups (**Supplementary Figure S3F**).

### 3.7 Enrichment analysis of the two key genes

**Figure 8:**
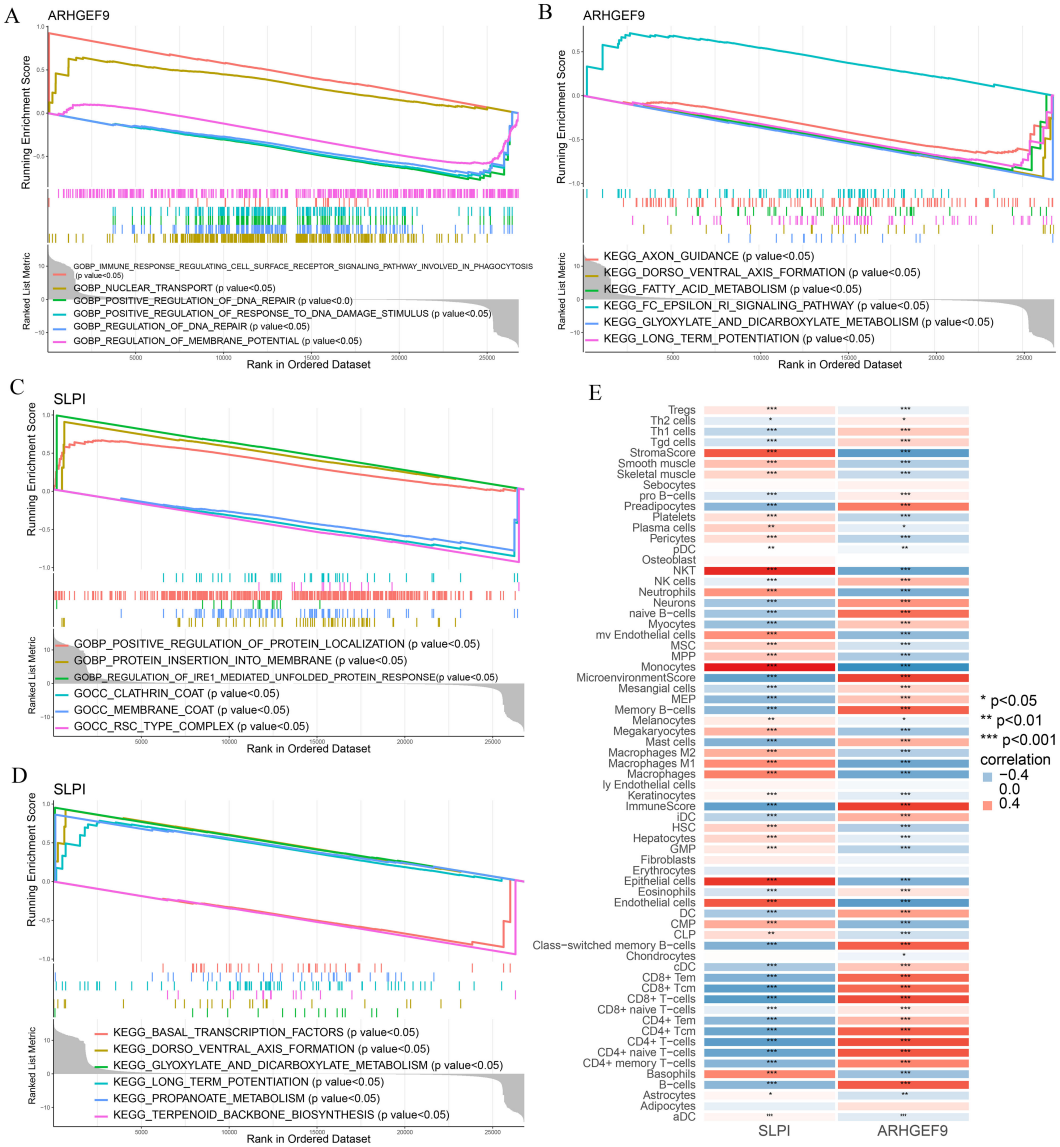
Functional enrichment and immune cell correlation analysis of characteristic genes in AP. **(A-D)** Enrichment biological functions and pathways of two hub genes identified by GSEA; **(E)** Immune cell correlation analysis of ARHGEF9 and SLPI. *P < 0.05; **P < 0.01; ***P < 0.001.

GSEA was used to determine the biological functions of the two key genes in AP and T2DM. According to the results of GSEA in the AP group, ARHGEF9 was significantly downregulated in pathways related to DNA regulation and metabolism (**Figures 8A, B**), whereas SLPI was significantly upregulated in pathways related to the regulation of protein response and localization (**Figures 8C, D**). Subsequently, we investigated the relationship between the two key genes and the immune environment of AP. The results showed that ARHGEF9 expression was significantly negatively correlated with the proportions of resting NK T cells, endothelial cells, and aDCs, whereas SLPI expression was significantly positively correlated with the proportions of epithelial cells, M1 and M2 macrophages, endothelial cells, and basophils (**Figure 8E**).

**Figure 9:**
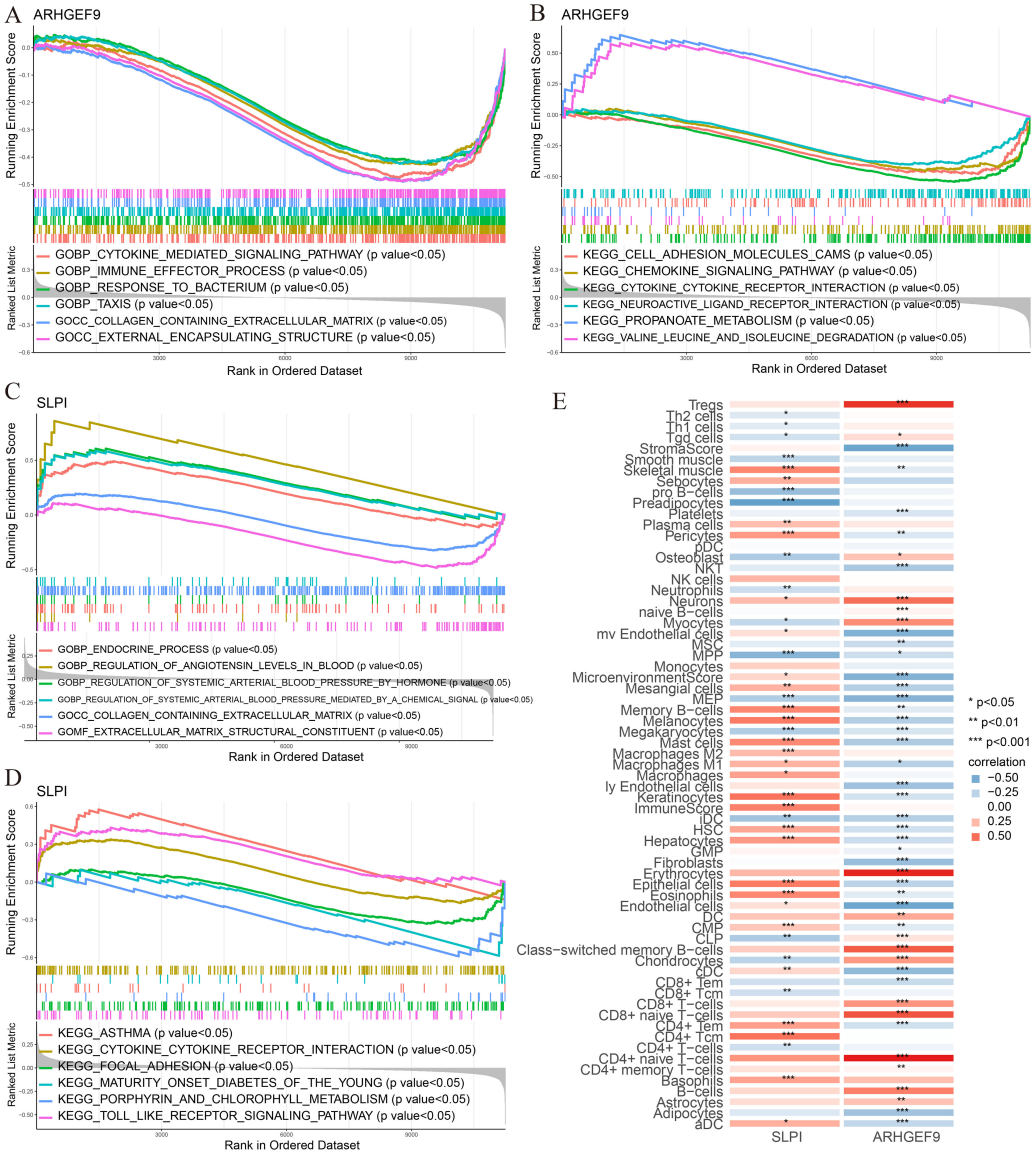
Functional enrichment and immune cell correlation analysis of characteristic genes in T2DM. **(A-D)** Enrichment biological functions and pathways of two hub genes identified by GSEA; **(E)** Immune cell correlation analysis of ARHGEF9 and SLPI. *P < 0.05; **P < 0.01; ***P < 0.001.

According to the results of GSEA in the T2DM group, ARHGEF9 was significantly downregulated in pathways related to amino acid metabolism and regulation (**Figures 9A, B**), whereas SLPI was significantly upregulated in pathways related to hormone regulation and cell interactions (**Figures 9C, D**). With regard to the relationship between the two genes and the immune environment of T2DM, ARHGEF9 expression was negatively correlated with the proportions of resting NK T cells, endothelial cells, aDCs, and mesangial cells, whereas SLPI expression was positively correlated with the proportions of epithelial cells, M1 macrophages, mast cells, and basophils (**Figure 9E**).

### 3.8 ceRNA networks, single-cell maps, and immunofluorescence analysis

**Figure 10:**
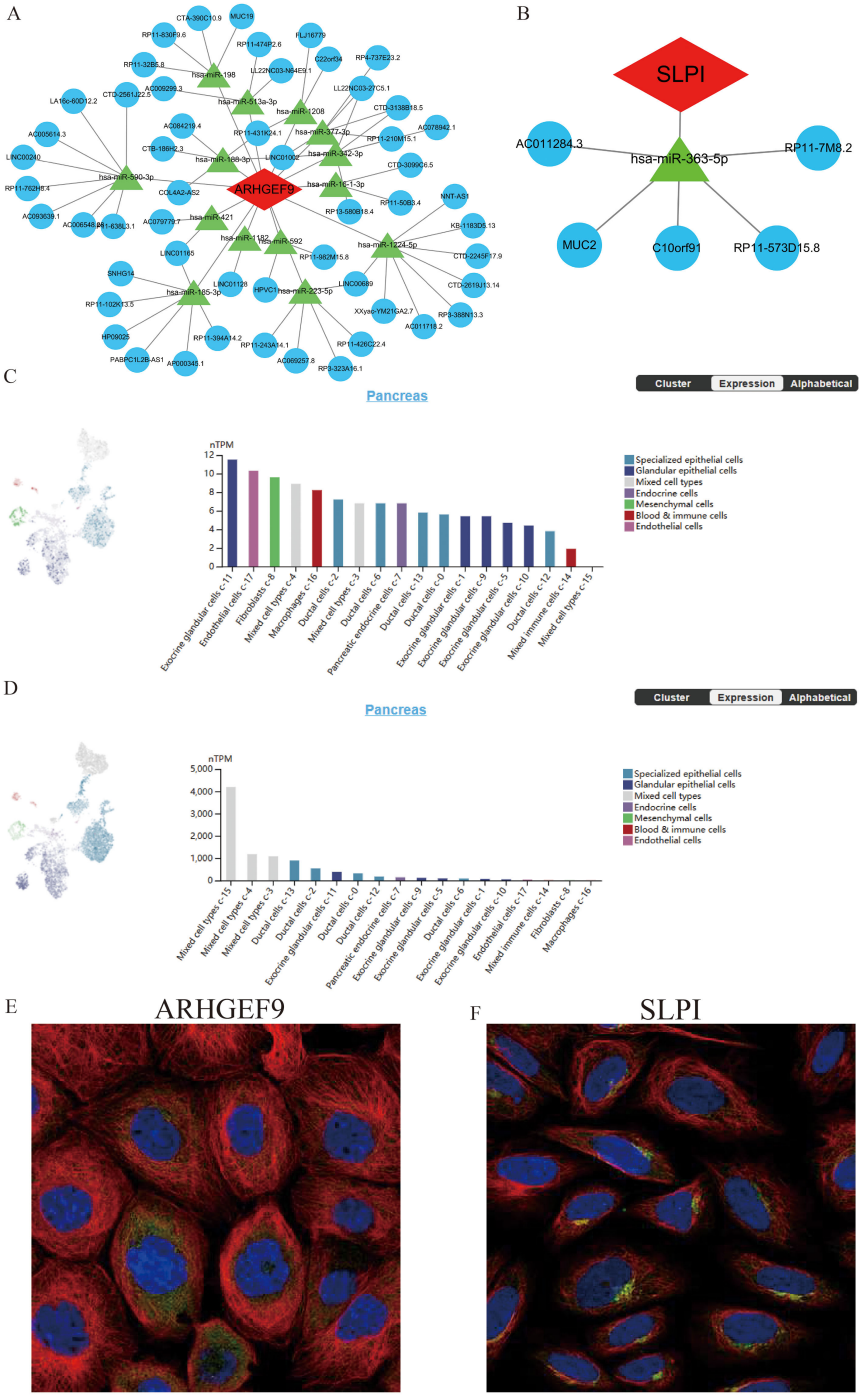
The ceRNA networks, single-cell maps, and immunofluorescence of ARHGEF9 and SLPI. **(A)** The ceRNA network of ARHGEF9; **(B)** The ceRNA network of SLPI; **(C)** The single-cell type atlases of ARHGEF9 in the pancreatic tissues; **(D)** The single-cell type atlases of SLPI in the pancreatic tissues; **(E)** The immunofluorescence of ARHGEF9 in cell line A-431, target protein (green), nucleus (blue), microtubules (red); **(F)** The immunofluorescence of SLPI in cell line SiHa, target protein (green), nucleus (blue), microtubules (red).

SLPI- and ARHGEF9-based ceRNA networks were separately constructed using various public databases. Eventually, 14 objective miRNAs and 47 objective lncRNAs interacting with ARHGEF9 (**Figure 10A**) and 1 objective miRNA and 5 objective lncRNAs interacting with SLPI (**Figure 10B**) were identified. The ceRNA networks constructed based on these miRNAs and lncRNAs revealed transcriptional regulatory mechanisms for the two genes. Furthermore, SLPI was found to be distributed primarily in exocrine gland cells and endothelial cells, whereas ARHGEF9 was found to be distributed primarily in duct cells (**Figures 10C, D**). With regard to their locations in cells, SLPI was detected in mitochondria, whereas ARHGEF9 was detected in mitochondria and endoplasmic reticulum (**Figures 10E, F**).

### 3.9 Identification of potential small-molecule compounds for the treatment of AP and T2DM

To identify potential drugs for the treatment of AP and T2DM, we imported the 14 upregulated DEGs common between AP and T2DM into the CMAP database for analysis. The top 10 small-molecule compounds are shown in **Table 1**. Notably, the tyrosine kinase inhibitor imatinib had the highest negative connectivity score. Subsequently, we performed molecular docking of these 10 small-molecule compounds with SLPI and ARHGEF9 (**Supplementary Figures S4**, **S5**). The minimum binding energies of all docked complexes are shown in **Table 1**. The minimum binding energies of complexes were typically less than -6.0 kcal/mol, which indicated that both proteins had a good binding affinity for the 10 small-molecule compounds. In particular, the SLPI–imatinib (-9.1 kcal/mol) and ARHGEF9–imatinib (-10.4 kcal/mol) complexes had the lowest free binding energies. These results suggest that imatinib is a promising therapeutic agent for both AP and T2DM.

**Table 1:** Potential treatment options for AP and T2DM analyzed by CMap and molecular docking.

Rank	CMap name	Moa	Norm_cs	FDR	Free binding energy (kcal/mol)	
					SLPI	ARHGEF9
1	imatinib	PDGFR inhibitor|Bcr-Abl inhibitor|KIT inhibitor	-2.1003	15.6536	-9.1	-10.4
2	procainamide	Sodium channel inhibitor	-2.0882	15.6536	-5.3	-6.2
3	simvastatin	HMGCR inhibitor	-2.0699	15.6536	-6.5	-9.1
4	BIBX-1382	EGFR inhibitor|Tyrosine kinase inhibitor	-2.0666	15.6536	-6.9	-8.9
5	physostigmine	Cholinesterase inhibitor|Acetylcholinesterase inhibitor	-2.051	15.6536	-6.2	-7.8
6	carbamazepine	Carboxamide antiepileptic	-2.0306	15.6536	-6.8	-9.5
7	phentermine	Dopamine receptor antagonist|Serotonin reuptake inhibitor	-2.0163	15.3525	-4.7	-6.0
8	TCPOBOP	CAR agonist	-2.0096	15.3525	-6.3	-8.4
9	rigosertib	Cell cycle inhibitor|PLK inhibitor	-2.0082	15.3525	-5.8	-7.5

## 4 Discussion

AP is an acute inflammatory condition of the pancreas that can lead to various severe symptoms (33). This condition often presents with serious comorbidities and is associated with a high mortality rate (34, 35). Fu et al. (36) showed that the mortality rate of AP was 3.8%, whereas that of severe AP was 16.3%. Currently, gallstones, alcoholism, and dyslipidemia are considered to be the major predisposing factors for AP (37). The pathological mechanisms underlying pancreatitis are complex owing to the wide range of potential etiological factors, including genetic, behavioral, and environmental factors, and the interactions between them (38). Therefore, an in-depth understanding of the pathogenesis of AP and early diagnosis and treatment are keys to reducing the morbidity and mortality rates of AP.

According to a survey conducted by the International Diabetes Federation (39), the number of individuals with DM is projected to increase from 536.6 million in 2021 to 783.2 million by 2045 worldwide, making it one of the most prevalent endocrine illnesses. T2DM is a chronic metabolic disease characterized by insulin resistance and elevated blood glucose levels (40). T2DM involves a number of complex pathophysiological mechanisms, and it has been found that high sugar intake and dysregulation of glucose and lipid metabolism may be a major contributing factor in the development of diabetes (41, 42). Notably, T2DM has similar pathogenic risk factors to AP such as hypertriglyceridemia and alcohol. T2DM has been shown to have a positive correlation with the risk of AP (43). A recent prospective study showed that 3%, 7%, 9%, and 11% of participants developed DM at 6, 12, 18, and 24 months after an AP episode, respectively (44). Despite these findings, information regarding the exact time during which endocrine dysfunction occurs after AP is lacking. Some studies have shown complete resolution or notable improvements over time, whereas others have shown persistent endocrine dysfunction (45–47). These findings suggest that dysregulation of glucose metabolism is common in patients with AP; however, it may be reversible. Moreover, according to an extensive cohort study, individuals with T2DM are more likely to develop AP than those without DM (48). An Asian population-based cohort study supported this finding, showing that individuals with DM had a 2-fold higher incidence of AP than those without DM (49). However, the mechanisms underlying the complex interplay between AP and T2DM remain unclear. In this study, we identified key genes associated with both AP and T2DM through bioinformatic analysis. The findings may improve early identification, treatment, and prevention of the two diseases.

Initially, we identified 26 common DEGs, including 14 upregulated and 12 downregulated genes, between AP and T2DM. To determine the potential mechanisms underlying the interaction between AP and T2DM, we used the Metascape database to perform functional and DisGeNET enrichment analyses of 26 DEGs. The results showed that the DEGs were involved in immune effector processes and blood vessel development and were closely related to hypertriglyceridemia and dyslipidemia, suggesting a relationship between lipid metabolism and the two diseases. In addition, subsequent MR analysis showed that dyslipidemia might serve as a link between AP and T2DM. AP is independently associated with hyperlipidemia, and hypertriglyceridemia-induced pancreatitis frequently presents with a severe course of illness (50–52). A large prospective cohort study found that cumulative exposure to hypertriglyceridemia was significantly associated with an increased risk of T2DM (53). Overproduction of large triglyceride-rich lipoproteins and impairment of catabolism are associated with insulin resistance, which contributes to hypertriglyceridemia frequently seen in patients with DM (54). DM is the most common secondary factor contributing to hypertriglyceridemia-induced pancreatitis. Patients with untreated or poorly managed DM have higher triglyceride levels, which increase the risk of pancreatitis (55). In a cohort study on patients with severe hypertriglyceridemia associated AP, 62% had T2DM, rising to 79% in patients with severe hypertriglyceridemia (56). In addition, severe hypertriglyceridemia was specifically associated with DM treated with insulin, which suggested that advanced or uncontrolled DM was the primary metabolic cause of hypertriglyceridemia-induced pancreatitis. Moreover, lower triglyceride levels are thought to be associated with a lower incidence of pancreatitis (50). On the contrary, hypertriglyceridemia and obesity increase the risk of developing T2DM and AP. Their existence before the onset of AP increases the likelihood of developing T2DM after AP and may accelerate the onset of overt T2DM (57). The findings of this study suggest that hypertriglyceridemia and dyslipidemia are key factors contributing to the development of AP and T2DM. Therefore, strategies aimed at preventing and treating dyslipidemia may help control or delay the development of AP and T2DM.

AP is diagnosed if at least two of the following three criteria are met: (1) typical abdominal pain; (2) serum lipase activity at least three times greater than the upper limit of normal; and (3) characteristic morphological findings on imaging (58). The diagnostic criteria for DM are as follows: typical diabetic symptoms and random blood glucose levels of ≥11.1 mmol/L or fasting blood glucose levels of ≥7.0 mmol/L or 2-h blood glucose levels of ≥11.1 mmol/L on OGTT or HbA1c levels of ≥6.5% (59). In the early stage of AP, abdominal pain is not evident and serum lipase levels often begin to increase after 24 hours of onset. Consequently, the diagnosis is delayed and the prognosis is affected. Early diagnosis and assessment of patients with AP may play a role in improving the prognosis and facilitate the development of novel clinical treatments for AP. The diagnosis of DM often relies on already elevated blood glucose levels; therefore, recognizing the onset of the disease early, before the blood glucose level increases, is particularly important for controlling disease development. In this study, we identified two key genes associated with both AP and T2DM (SLPI and ARHGEF9) using several machine learning algorithms and developed a 2-gene diagnostic model. Subsequently, we used multiple analytical methods to evaluate the predictive performance of the diagnostic model in AP and T2DM. Specific expression patterns of ARHGEF9 and SLPI were observed in AP and T2DM, and both genes were found to have high diagnostic value in the two diseases. These findings highlight the genetic similarity between AP and T2DM. These results suggest that ARHGEF9 and SLPI are promising diagnostic biomarkers for AP and T2DM, which may facilitate early diagnosis and prompt treatment in clinical settings.

The SLPI gene is an important regulator of innate and acquired immunity and controls the growth of the gut microbiota (60). The ARHGEF9 gene is involved in the growth and development of cranial nerves (61) and has been shown to play a role in inhibiting the growth of both hepatocellular carcinoma (62) and gastric cancer (63) cells. However, the functions of ARHGEF9 and SLPI in AP and T2DM and the common mechanisms and pathways involved in the development of the two diseases remain unclear. In this study, we performed an in-depth analysis to investigate the potential roles and mechanisms of SLPI and ARHGEF9 in AP and T2DM. The results showed that in both AP and T2DM, ARHGEF9 was significantly downregulated in pathways related to DNA regulation, fatty acid metabolism, cytokine–cytokine receptor interactions, and chemokine signaling, whereas SLPI was significantly enriched in pathways related to the regulation of protein response and localization, glyoxylate and dicarboxylate metabolism, and cytokine–cytokine receptor interactions. Regarding the relationship between the two genes and the immune environment of the two diseases, ARHGEF9 expression was significantly negatively correlated with the proportions of resting NK T cells, endothelial cells, and aDCs, whereas SLPI expression was significantly positively correlated with the proportions of epithelial cells, M1 macrophages, and basophils.

Finally, we found that SLPI and ARHGEF9 serve as potential therapeutic targets for AP and T2DM and that imatinib may inhibit disease progression by targeting these genes. Imatinib has been shown to delay the development of diabetes and induce remission of diabetes in non-obese diabetic mice (64). The safety and efficacy of imatinib in the treatment of type 1 diabetes mellitus have been assessed in a clinical study. The findings indicated that participants in the imatinib group required less insulin and had lower HBA1c levels during treatment than those in the placebo group; however, these effects subsided after the treatment ended. Imatinib may improve peripheral insulin sensitivity and beta-cell activity, which may account for its metabolic effects (65). Furthermore, inhibition of discoidin domain receptors by imatinib has been shown to prevent pancreatic fibrosis in a mouse model of chronic pancreatitis (66). Therefore, imatinib is a promising drug for the treatment of AP and T2DM as well as to inhibit the correlation between the two diseases.

To the best of our knowledge, this study is the first to elucidate the relationship between T2DM and AP and identify common genes involved in the development of both diseases through comprehensive bioinformatic analysis. However, this study has some limitations that should be acknowledged. First, the limited dataset, small sample size, and lack of clinical information might not have adequately represented the characteristics of the target population. Therefore, large-sample multi-center prospective randomized controlled trials should be conducted to validate the predictive efficacy of the 2-gene diagnostic model developed in this study. Second, owing to the limitations of the datasets used in this study, we could not differentiate between patients with T2DM with or without AP and patients with AP with or without T2DM. Consequently, we could not assess the significance of the diagnostic model in predicting that patients with AP may have concurrent T2DM or patients with T2DM may have concurrent AP. Third, additional experiments are warranted to validate dyslipidemia as the link between AP and T2DM and to investigate the mechanisms of SLPI and ARHGEF9 in AP and T2DM and their relationship with dyslipidemia.

## 5 Conclusion

Our study identified dyslipidemia as a possible common mechanism of T2DM and AP and constructed a two-gene diagnostic model for early recognition of T2DM and AP through a series of machine learning approaches. Most importantly, we found that imatinib may be a potential treatment for T2DM and AP.

## Data Availability

The datasets analyzed in this work may be found in the GEO database (https://www.ncbi.nlm.nih.gov/gds) and the IEU OPEN GWAS (https://gwas.mrcieu.ac.uk/). Further inquiries can be directed to the corresponding authors.

## Supplementary material

Supplementary Table 1 MR analysis results of HDL-C, LDL-C, TG, and APOA-1 on T2DM and AP.
