# Supplementary material for: Exploring the pathogenesis, biomarkers, and potential drugs for type 2 diabetes mellitus and acute pancreatitis through a comprehensive bioinformatic analysis
Source: Front Endocrinol (Lausanne). 2024 Nov 20;15:1405726. doi: 10.3389/fendo.2024.1405726 (PMC11614670; doi:10.3389/fendo.2024.1405726)
Supplement: Supplementary file 2 [file Image1.pdf]

A

Exposure	Method	nSNP	Pval	OR(95% CI)
HDL cholesterol	MR Egger	75	0.0566	0.7873(0.618-1.0028)
HDL cholesterol	Weighted median	75	4.890175e-05	0.7035(0.5936-0.8336)
HDL cholesterol	Inverse variance weighted	75	1.569442e-09	0.6865(0.6075-0.7756)
HDL cholesterol	Simple mode	75	0.033	0.6926(0.4973-0.9647)
HDL cholesterol	Weighted mode	75	0.0011	0.7187(0.5944-0.869)
LDL cholesterol	MR Egger	78	0.9115	1.0101(0.847-1.2046)
LDL cholesterol	Weighted median	78	0.4865	1.0485(0.9176-1.1981)
LDL cholesterol	Inverse variance weighted	78	0.4899	1.0396(0.9312-1.1606)
LDL cholesterol	Simple mode	78	0.9612	1.0061(0.7889-1.283)
LDL cholesterol	Weighted mode	78	0.5167	1.0421(0.9206-1.1795)
Triglycerides	MR Egger	45	0.0603	1.2293(0.9967-1.5162)
Triglycerides	Weighted median	45	0.0014	1.3401(1.1198-1.6037)
Triglycerides	Inverse variance weighted	45	1e-04	1.3086(1.1421-1.4993)
Triglycerides	Simple mode	45	0.0486	1.4166(1.0119-1.9832)
Triglycerides	Weighted mode	45	6e-04	1.3427(1.1494-1.5685)
apolipoprotein A-I	MR Egger	273	0.6509	0.9541(0.7785-1.1692)
apolipoprotein A-I	Weighted median	273	0.354	0.9288(0.7945-1.0858)
apolipoprotein A-I	Inverse variance weighted	273	7e-04	0.8011(0.7046-0.9108)
apolipoprotein A-I	Simple mode	273	0.7258	0.932(0.6289-1.3811)
apolipoprotein A-I	Weighted mode	273	0.6581	0.9619(0.81-1.1422)

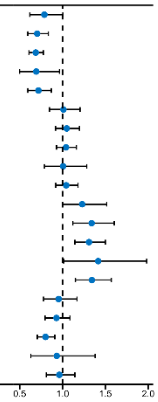

B

Exposure	Method	nSNP	Pval	OR(95% CI)
HDL cholesterol	MR Egger	45	0.148	0.9993(0.9984-1.0002)
HDL cholesterol	Weighted median	45	0.1131	0.9993(0.9984-1.0002)
HDL cholesterol	Inverse variance weighted	45	0.0269	0.9993(0.9986-0.9999)
HDL cholesterol	Simple mode	45	0.2938	0.999(0.997-1.0009)
HDL cholesterol	Weighted mode	45	0.0326	0.9992(0.9984-0.9999)
LDL cholesterol	MR Egger	32	0.5069	0.9993(0.9971-1.0014)
LDL cholesterol	Weighted median	32	0.8275	0.9998(0.9984-1.0013)
LDL cholesterol	Inverse variance weighted	32	0.5257	0.9997(0.9987-1.0007)
LDL cholesterol	Simple mode	32	0.618	0.9994(0.997-1.0018)
LDL cholesterol	Weighted mode	32	0.7791	0.9998(0.9983-1.0012)
Triglycerides	MR Egger	35	0.0294	0.9979(0.9961-0.9997)
Triglycerides	Weighted median	35	0.1312	0.9989(0.9975-1.0003)
Triglycerides	Inverse variance weighted	35	0.5352	0.9997(0.9988-1.0006)
Triglycerides	Simple mode	35	0.9999	1(0.9975-1.0025)
Triglycerides	Weighted mode	35	0.0368	0.9985(0.9971-0.9998)
apolipoprotein A-I	MR Egger	116	0.6951	0.9997(0.9983-1.0011)
apolipoprotein A-I	Weighted median	116	0.7976	0.9998(0.9984-1.0012)
apolipoprotein A-I	Inverse variance weighted	116	0.0232	0.999(0.9982-0.9999)
apolipoprotein A-I	Simple mode	116	0.5883	0.9992(0.9965-1.002)
apolipoprotein A-I	Weighted mode	116	0.3874	0.9994(0.9982-1.0007)

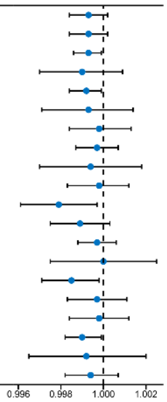

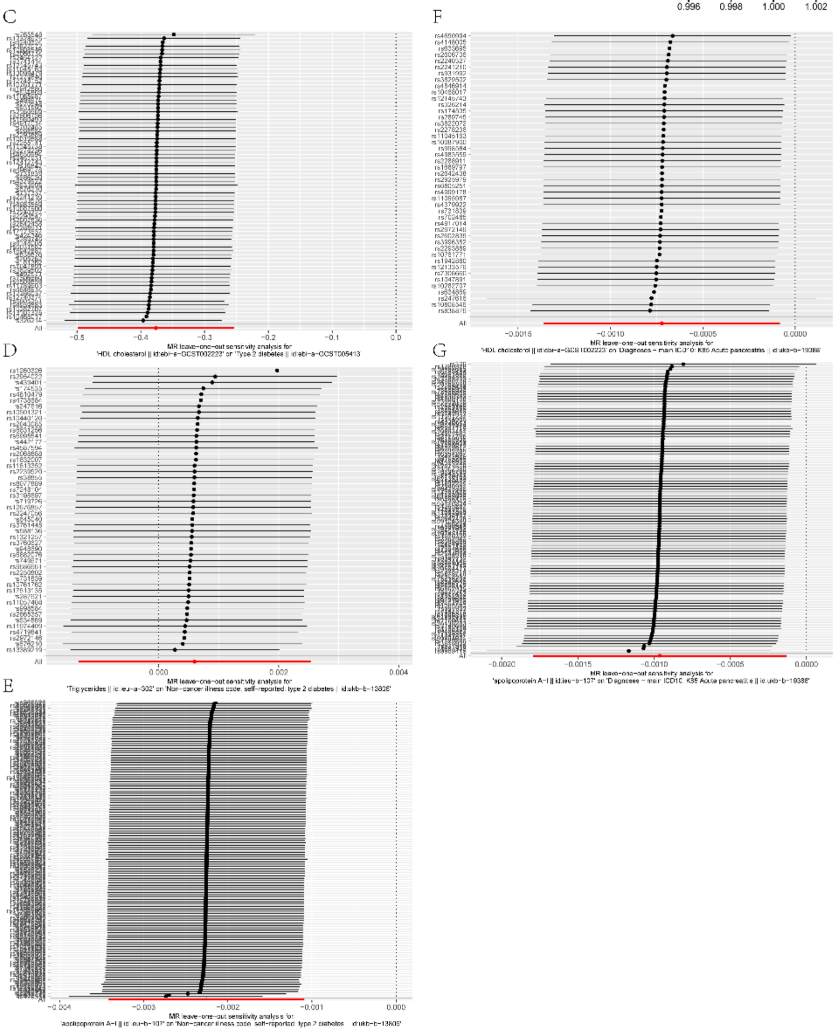

Figure S1. Forest plot of MR analysis results for different HDL-C, LDL-C, TG, and APOA-I on (A) T2DM and (B) AP; Forest plot of the leave one out results of this study: (C) HDL-C and T2DM, (D)TG and T2DM, (E) Apoa-I and T2DM, (F)HDL-C and AP, (G)Apoa-I and AP.

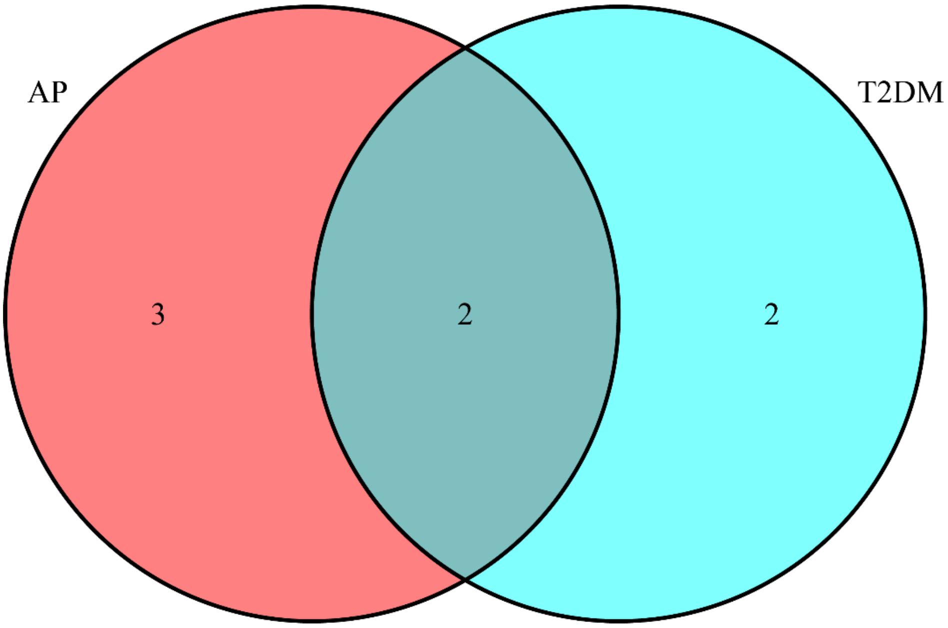

Figure S2. Common DEGs for AP and T2DM.

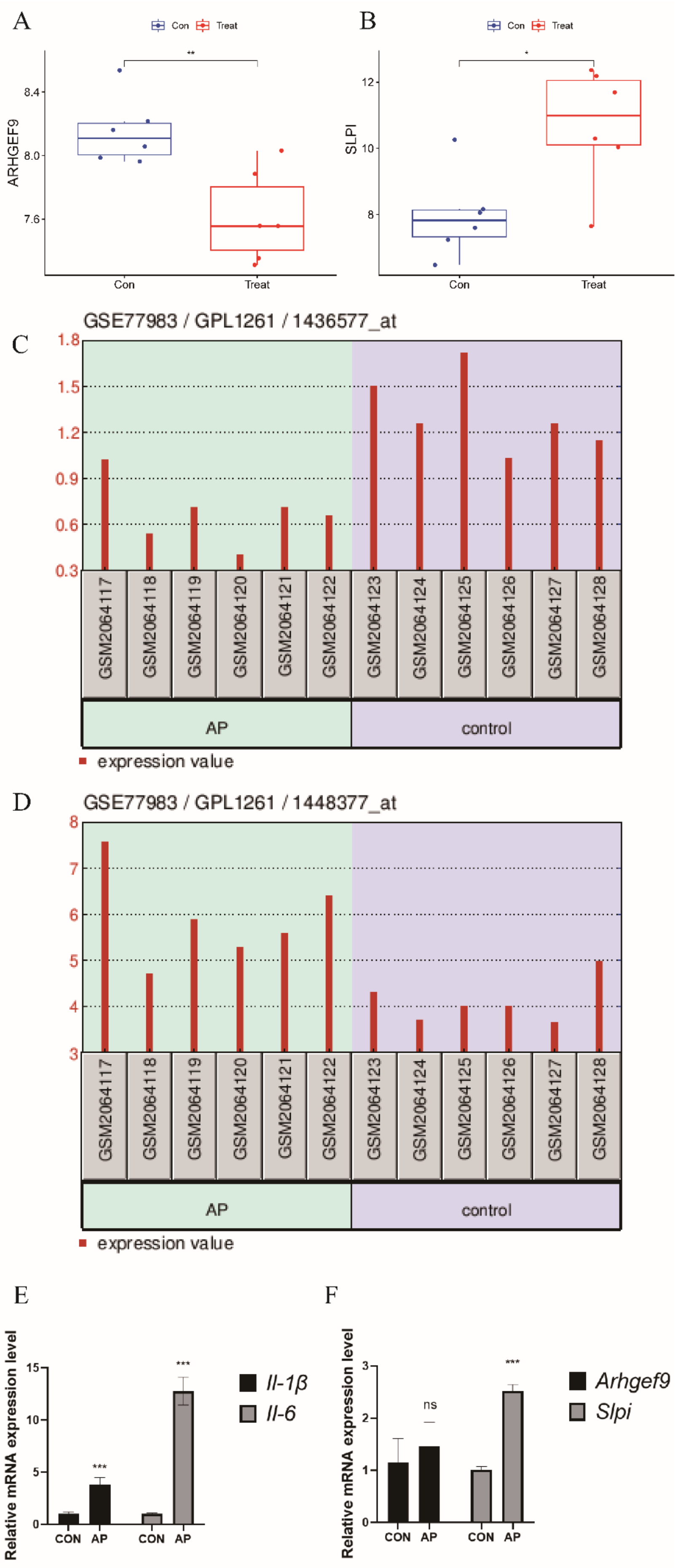
A
Con
Treat
**
ARHGEF9
8.4
8.0
7.6
B
*
SLPI
12
10
8
C
GSE77983 / GPL1261 / 1436577_at
1.8
1.5
1.2
0.9
0.6
0.3
GSM2064117
GSM2064118
GSM2064119
GSM2064120
GSM2064121
GSM2064122
GSM2064123
GSM2064124
GSM2064125
GSM2064126
GSM2064127
GSM2064128
AP
control
expression value
D
GSE77983 / GPL1261 / 1448377_at
8
7
6
5
4
3
E
Relative mRNA expression level
15
10
5
0
CON
AP
***
Il-1β
Il-6
F
3
2
1
ns
Arhgef9
Slpi

Figure S3. Validation of two biomarkers for T2DM and AP. (A) The box plot showed the difference in ARHGEF9 expression between the T2DM and the normal samples; (B) The box plot showed the difference in SLPI expression between the T2DM and the normal samples; (C) The expression of ARHGEF9 in the AP and the normal samples; (D) The expression of SLPI in the AP and the normal samples; (E) Expression levels of inflammatory factors in cell lines; (F) Expression levels of core genes in cell lines.

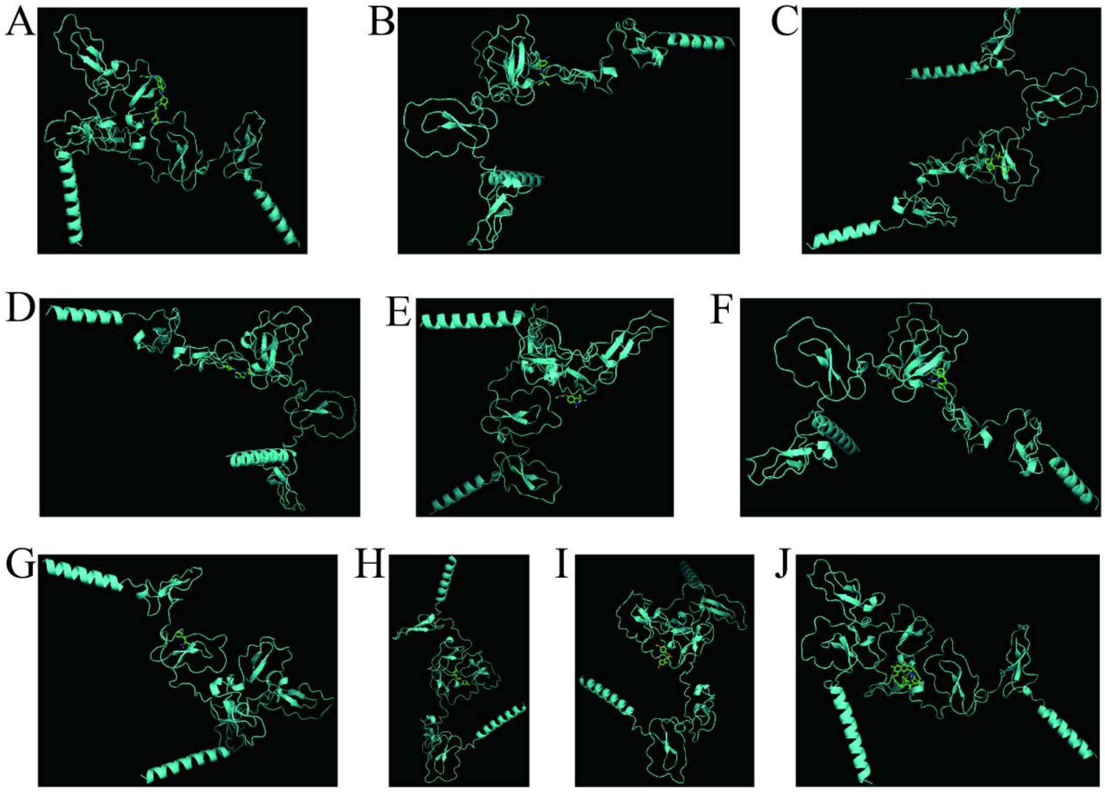

Figure S4. Molecular docking experiment of 10 small molecule compounds with SLPI. (A) imatinib; (B) procainamide; (C) simvastatin; (D)BIBX-1382; (E) physostigmine; (F) carbamazepine; (G) phentermine; (H) TCPOBOP; (I) rigosertib; (J) rifapentine.

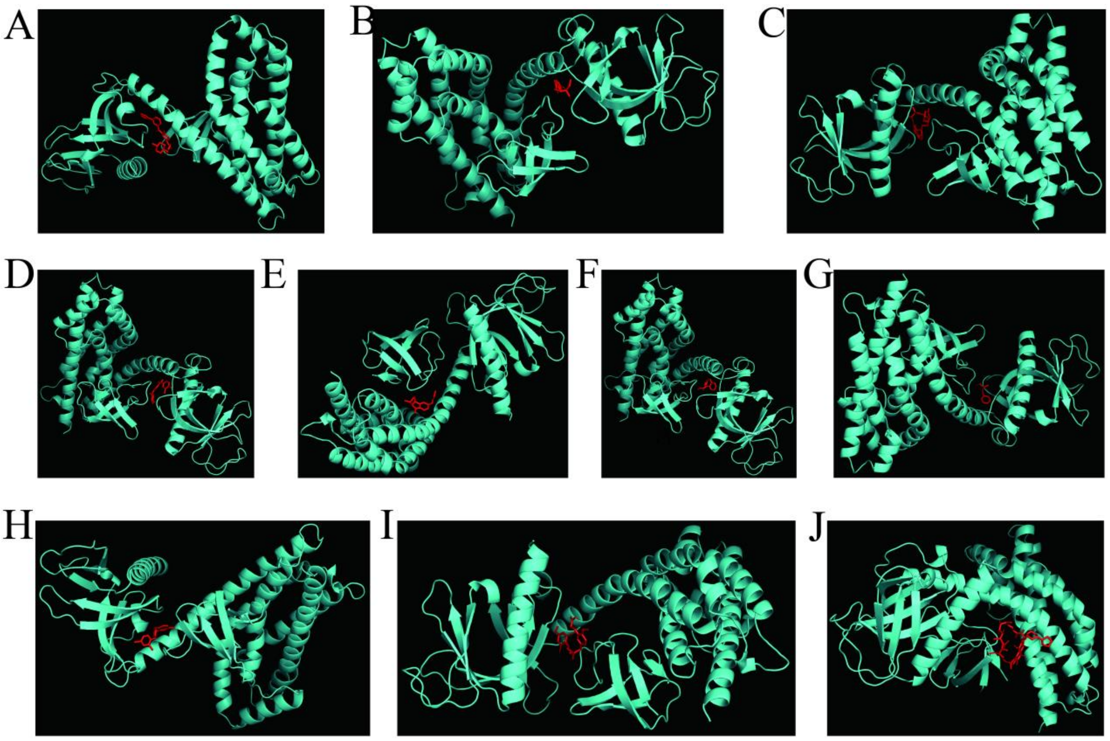

Figure S5. Molecular docking experiment of 10 small molecule compounds with ARHGEF9. (A) imatinib; (B) procainamide; (C) simvastatin; (D)BIBX-1382; (E) physostigmine; (F) carbamazepine; (G) phentermine; (H) TCPOBOP; (I) rigosertib; (J) rifapentine.
